# One-Step Differential Detection of OXA-48-Like Variants Using High-Resolution Melting (HRM) Analysis

**DOI:** 10.3390/antibiotics9050256

**Published:** 2020-05-15

**Authors:** Min Yi Lau, Kartini Abdul Jabar, Kek Heng Chua, Boon Pin Kee, Sasheela Sri La Sri Ponnampalavanar, Chun Wie Chong, Cindy Shuan Ju Teh

**Affiliations:** 1 Department of Medical Microbiology, Faculty of Medicine, University of Malaya, Kuala Lumpur 50603, Malaysia; minyi@um.edu.my (M.Y.L.); kartini.abduljabar@ummc.edu.my (K.A.J.); 2 Department of Biomedical Science, Faculty of Medicine, University of Malaya, Kuala Lumpur 50603, Malaysia; khchua@um.edu.my (K.H.C.); bpkee@um.edu.my (B.P.K.); 3 Department of Infectious Diseases, University Malaya Medical Centre, Kuala Lumpur 50603, Malaysia; sheela@ummc.edu.my; 4 School of Pharmacy, Monash University Malaysia, Subang Jaya 47500, Malaysia; chong.chunwie@monash.edu

**Keywords:** carbapenem-resistant *Klebsiella pneumoniae*, *Enterobacteriaceae*, OXA-48, HRM, real-time PCR

## Abstract

OXA-48-like carbapenemase gene remains a hidden threat, as different OXA-48 variants have varying presentations of susceptibility to antibiotics that might affect the treatment decisions. Rapid detection and differentiation of OXA-48-like carbapenemase genes are critical for targeted treatment and infection control. In this study, we aimed to develop high-resolution melting (HRM) analysis for the differentiation of OXA-48 variants. HRM analysis is a post-polymerase chain reaction (post-PCR) method for identification of small variations in nucleic acid sequences based on the PCR dissociation curve. A total of 82 bacterial strains, which consisted of *Enterobacteriaceae* and non-*Enterobacteriaceae,* were collected from a tertiary teaching hospital. The sensitivity and specificity of the assay were determined, and the developed assay was evaluated using the collected isolates against conventional-sequencing method. Overall, the developed assay was able to detect isolates that harboured OXA-48 and OXA232/OXA-181 by showing two distinct peaks at 81.1 ± 0.2 °C and 82.1 ± 0.2 °C, respectively. The detection limit of the assay was 1.6 x 10^−6^ ng/µL for OXA-48 and 1.8 × 10^−7^ ng/µL for OXA-232/OXA-181. This assay showed 100% specificity when evaluated on a panel of 37 isolates comprised of different species of bacteria and yeasts. When the assay with isolates collected in the year 2016 was first evaluated, the assay showed comparable results with conventional PCR-sequencing method where 34 OXA-48 and OXA-232/OXA-181 were detected. By using HRM analysis, the presence of OXA-48-like variants could be easily identified within 3 h from the pure culture.

## 1. Introduction

The prevalence of carbapenem-resistant *Enterobacteriaceae* (CRE), especially those harbouring OXA-48-like carbapenem hydrolysing class D β-lactamases, has increased at an alarming rate [1]. OXA-48 is a plasmid borne enzyme that is only detected in *Enterobacteriaceae,* and is commonly associated with *Klebsiella pneumoniae* (*K. pneumoniae*) [1]. The first OXA-48 carbapenemase gene was identified in a *K. pneumoniae* isolate from Istanbul, Turkey in 2001 and subsequently disseminated in many countries including India, North Africa, and Europe [1,2].

In the past decade, several OXA-48-like variants have been described such as OXA-48, OXA-232, OXA-181, OXA-162, OXA-163, OXA-244, and OXA-245 [3]. Each variant differs from OXA-48 by one to five amino acid substitution or deletion, which results in different hydrolytic profiles. For example, OXA-232 exhibited five amino acid substitutions compared to OXA-48 but differed from OXA-181 by just one point mutation [1]. However, not all OXA-48-like variants have been reported in Asia. The most common detected OXA-48-like variants in Asia are OXA-48, OXA-232, and OXA-181. In Malaysia, only OXA-48 and OXA-232 were previously reported [4,5]. Nonetheless, recent studies showed that the cases caused by OXA-48 carbapenemase gene had increased drastically [4,5].

OXA-48 hydrolysed penicillin at a high level but was low in carbapenem, and it showed weak activity against expanded-spectrum cephalosporin [1]. On the other hand, OXA-181 showed higher ability to hydrolyse carbapenems, while OXA-232 possessed lower hydrolytic ability to carbapenems as compared to OXA-48 and OXA-181 [6]. Furthermore, OXA-232 hydrolysed all penicillin (with the exception of temocillin) more efficiently than OXA-48 and OXA-181 [7]. This suggested that different OXA-48-like variants have different presentation of susceptibilities to antibiotics, which might affect the treatment decisions. Rapid and accurate detection of OXA-48-like carbapenemases are vital to control their dissemination. The current method for differential detection of OXA-48-like variants harboured by the carbapenem-resistant *Enterobacteriaceae* is by polymerase chain reaction (PCR), followed by direct sequencing to identify the point of mutations. Due to the high level of similarity among OXA-48-like variants, DNA sequencing is the currently preferred method to identify OXA-48 variants with high accuracy. However, the major drawback of the method is long turn-around time. High-resolution melting (HRM) analysis has been used for rapid detection of nucleotide polymorphisms within PCR products based on their melting curve. This method is shown to be sensitive and specific [8]. To address the limitation of conventional method, we have developed a HRM assay for rapid detection and differentiation of OXA-48-like carbapenamase gene.

## 2. Results

### 2.1. Detection and Differentiation of OXA-48 and OXA-232/OXA-181 by Real-Time PCR with HRM Analysis

**Figure 1:**
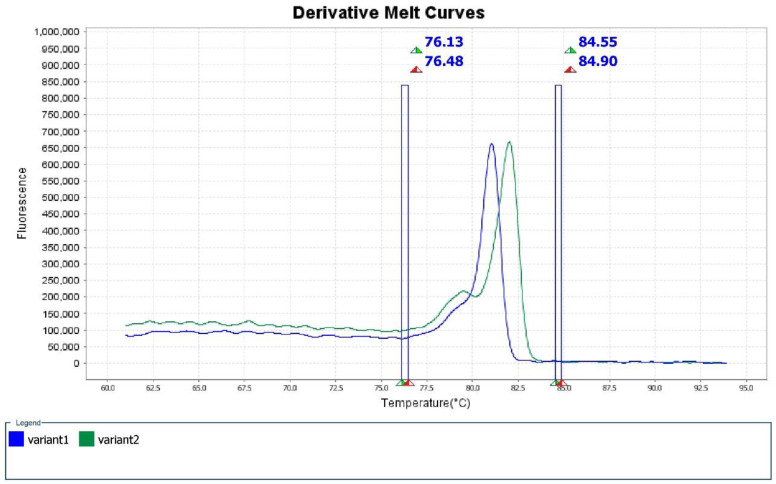
Derivative melt curves of OXA-48 (blue) and OXA-232/OXA-181 (green). The Tm values for variant 1 and variant 2 were 81.1 ± 0.2 °C and 82.1 ± 0.2 °C respectively.

**Figure 2:**
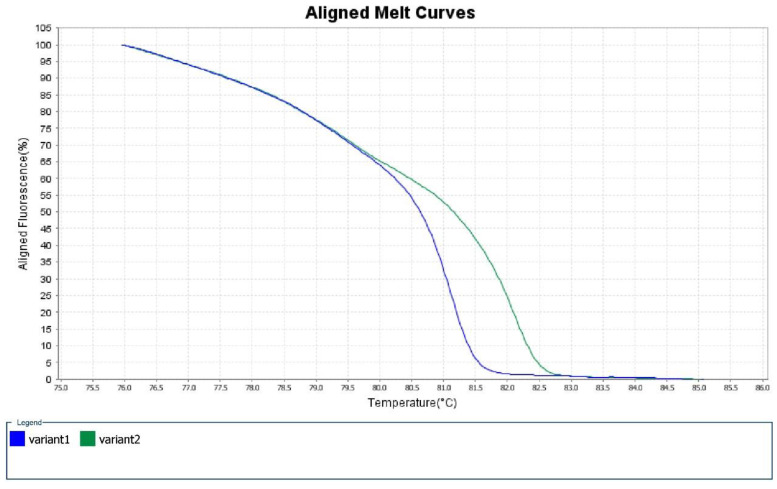
Aligned melt curves of OXA-48 (blue) and OXA-232/OXA-181 (green).

In the 298-bp region amplified by the designed primers, OXA-48 differs from OXA-181 and OXA-232 by four and five amino acids substitutions, respectively, while OXA-181 and OXA-232 only differ by one nucleotide substitution at position 642 (A to T). The derivative of melt curves produced by HRM software showed two distinct peaks (Figure 1), representing OXA-48 and OXA-232/OXA-181 at temperature of 81.1 ± 0.2 °C and 82.1 ± 0.2 °C, respectively. The aligned melt curves were shown in Figure 2. No amplification was observed for the negative control (CRE-192).

### 2.2. Sensitivity and Specificity Test

**Figure 3:**
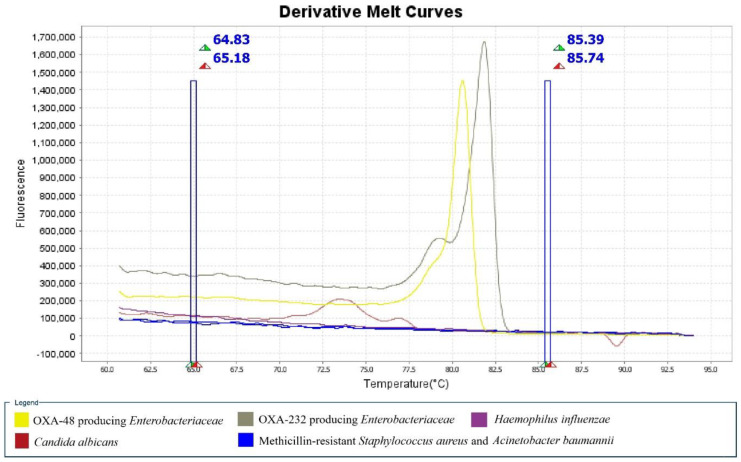
Derivative melt curve of OXA-48-like carbapenemase gene detection from different species. Amplification peak was observed from *Enterobacteriaceae*, which carried OXA-48 (yellow) and OXA-232 (grey) carbapenemase gene, whereas no peak was showed from other species including *Candida albicans*, *Haemophilus influenzae*, *Staphylococcus aureus*, and *Acinetobacter baumannii*, which do not produce OXA-48-like carbapenemase gene.

The detection limit of OXA-48 was found to be 1.6 × 10^−6^ ng/µL, while OXA-232/OXA-181 was 1.8 × 10^−7^ ng/µL. This newly developed assay successfully detected OXA-48-like variants from all 17 *Enterobacteriaceae.* No amplification was observed for Methicillin-resistant *Staphylococcus aureus* (MRSA), *Candida albicans*, *Haemophilus influenzae,* and *Acinetobacter baumannii* (Figure 3). This assay successfully differentiated the OXA-232-producing *E. coli* isolate from other OXA-48 isolates and yielded 100% specificity. These observations suggested that this newly developed HRM assay was effective in identifying the presence of OXA-48-like gene and differentiating common OXA-48-like variants in CRE (OXA-48 and OXA-232/OXA-181).

### 2.3. Evaluation of Real-Time PCR assay with HRM Analysis Using CRE Isolates in 2016

Among the 45 isolates collected in 2016, 34 harboured OXA-48-like gene based on real-time PCR assay with HRM analysis and conventional PCR. According to the HRM analysis, the OXA-48 positive isolates were further resolved into two variants based on the two peaks observed at 81.1 ± 0.2 °C (31 isolates) and 82.1 ± 0.2 °C (3 isolates). All the 31 isolates were OXA-48, whereas the three isolates were OXA-181/ OXA-232 (Table 1). This finding was in agreement with sequencing results where three of the isolates carried different amino acids at position 104, 110, 175, 179, and 222 than other isolates.

Overall, the detection time required for HRM analysis from pure culture to the final results is about three hours, which is great improvement from the conventional PCR-sequencing method, which requires turnaround time of three days.

## 3. Discussion

CRE was frequently reported across Asia-Pacific region. In the past decade, OXA-48-like carbapenemase gene was the most prevalent in Europe, Mediterranean, and North Africa [9]. Recently, OXA-48-like carbapenemase gene has emerged and spread in Southeast-Asia, including Malaysia [5], Singapore [10], Brunei [11], Thailand [12], and Vietnam [13]. Here, we identified the prevalence of OXA-48-like carbapenemase gene in Malaysia in recent years. OXA-48 has been the most common carbapenemase gene detected in our hospital. The first report of OXA-48-like carbapenemase gene from our hospital was in 2013 [5]. Among forty-five CRKP strains investigated in this study, 75.5% harboured *bla*_OXA-48-like_. Although OXA-48-like carbapenemases has weak hydrolytic ability towards carbapenem, high level of carbapenem resistance may occur when combined with other mechanisms such as loss of porin on the outer membrane [9]. Furthermore, it has been reported that different OXA-48-like variants present different susceptibility to antibiotics [6]. Therefore, rapid detection of OXA-48 and its variants is important to control their dissemination and provides a reference for better treatment decision.

Phenotypic tests have been used to detect carbapenem resistance. However, OXA-48 and its variants have different susceptibility towards broad-spectrum cephalosporins and carbapenem [1]. Identification of OXA-48-like carbapenemase by phenotypic assay was difficult to interpret and most likely was underestimated due to the low level resistantance to carbapenem [9]. On the other hand, conventional phenotypic tests were time-consuming and low in sensitivity and specificity in OXA-48 detections [14]. Therefore, molecular techniques, particularly PCR, remain as the gold standard for identification of OXA-48 [14,15]. PCR screening for OXA-48 has been reported in several studies either using single targeted PCR assays or multiplex PCR assays together with detection of other carbapenemase genes, but not in differentiation of OXA-48-like carbapenemase gene variants [2,16,17]. Commercial kit such as Check-Direct carbapenemase-producing *Enterobacteriaceae* (CPE) assays (Check-Points, Wageningen, The Netherlands), Xpert Carba-R (Cepheid, Sunnyvale, CA, USA), EazyPlex Superbug ID complete A/B (Amplex, Giessen, Germany), and the very recent point-of-care GenePOC technology (GenePOC, Quebec City, QC, Canada) have also been developed and widely used for detection of carbapenemase gene (KPC, NDM, VIM, IMP-1, and OXA-48) [18,19,20,21]. However, none of these assays could differentiate OXA-48 variants. Indeed, single nucleotide mutation among OXA-48 and its variants are indistinguishable using conventional PCR.

In this study, our newly developed real-time PCR assay with HRM analysis not only detected the presence of OXA-48, the method also differentiated OXA-48 from OXA-232/OXA-181 based on the melt curve pattern. HRM analysis is frequently used for genotyping and detection of single nucleotide polymorphisms [22]. It has been used as a tool for rapid detection and differentiation of carbapenemase gene variants such as KPC gene variants [23,24]. More recently, Hemarajata et al. have developed a similar real-time PCR assay with HRM analysis to detect and differentiate OXA-48-like variants [25]. Although the assay developed by Hemarajata et al. could differentiate OXA-232 and OXA-181 from other OXA-48-like genes, the use of LunaProbe has directly increased the cost of test. As compared to our newly developed HRM assay, the cost of our assay will be cheaper and affordable for most of the molecular laboratory. In general, HRM analysis is less tedious and more rapid compare to conventional PCR-sequencing method. Simpler procedure and lesser step could significantly reduce cross contamination and human error. However, as HRM utilized specific primers that targeted flanking region of the targeted region (usually less than 250 base pair), it might not be able to capture new mutations that fall outside the targeted regions. In contrast, PCR-sequencing that does not have limitations on product size could detect the new mutation more efficiently.

The OXA HRM assay has demonstrated high sensitivity and specificity in detecting OXA-48-like carbapenemase gene in all previously characterized OXA-48-like isolates. In addition, this assay was specific in the detection of OXA-48-like carbapenemases. For instance, other OXA-type carbapenemases such as OXA-23-like carbapenemases in *Acinetobacter baumannii* did not yield positive result. For differentiation of the three most prevalent OXA-48 variants, only two melting curves were shown. The nucleic acid sequences of OXA-232 and OXA-181 only differ by a single base pair at position 642 (A to T), an amino acid mutation, causing the melting curve incomparable [7]. However, sequencing is still required needed to distinguish OXA-232 and OXA-181 since they have different hydrolytic profiles, and this has been identified as the limitation in the study.

Before the development of HRM analysis assay, differentiation of OXA-48-like carbapenemase gene variants was usually done by sequencing after PCR amplification, which required more than two days to obtain the results. HRM analysis has significantly shortened the process of analysing OXA-48-like variants into three hours. In addition, direct characterization of OXA-48-like carbapenemase variants in a single closed system has reduced the risk of contamination. However, we acknowledge that the lack of other OXA-48-like variants isolates to validate this newly developed assay is the major caveat of this study.

## 4. Materials and Methods

### 4.1. Ethics Approval

This study has been approved by UMMC Medical Ethics Committee (MEC ID: 20154-1249).

### 4.2. Bacterial Strains

All bacterial strains were isolated from various clinical specimens such as blood, urine, pus, tissue, fluid, sputum, and swab samples from a tertiary teaching hospital. They were revived and checked for purity before commencement of benchwork. All the isolates were then subjected to *bla*_OXA-48_ genes detection by PCR with reported primers sequences [2].

### 4.3. Development of Real-Time PCR Assay with HRM Analysis

OXA-48-like sequences were retrieved from the NCBI database as a reference for primers design. A set of primers that amplified 298-bp region of the conserved region flanking the variable sites was designed and checked by Primer BLAST. Real-time PCR with HRM analysis was performed in 20 µL reaction, which contained 10 µL of MeltDoctor™ HRM Master Mix (Applied Biosystems Inc, Waltham, MA, USA), 0.3 µM of primer HRM OXA-48 F (5′-TCGGGCAATGTAGACAGTTT-3′), 0.3 µM of primer HRM OXA-48 R (5′-GCCCTAAACCATCCGATGTG-3′), and 1 µL of template DNA (~20ng/µL). The real-time PCR with HRM was carried out in a 7500 Fast real-time PCR system (Applied Biosystems Inc, Waltham, MA, USA). The cycling parameter was set at 95 °C for 10 min (enzyme activation), followed by 40 cycles of amplification, including 95 °C for 15 s (denature) and 60 °C for 1 min (annealing). Next, melting step or dissociation step were carried out at 95 °C for 10 s (denature), 60 °C for 1 min (annealing), 95 °C for 15 s (high-resolution melting), and final annealing at 60 °C for 15 s. The melting curve profile was recorded and analyzed using High Resolution Melt software for Windows^®^ version 3.0.1. The condition for HRM analysis was tested by using the two control strains (CRE-48 and CRE-232, which harboured OXA-48 and OXA-232, respectively). One non-OXA-48 producer (CRE-192) was used as the negative control. The presence of OXA-48-like carbapenemase genes was identified based on the melt curve pattern.

### 4.4. Sensitivity and Specificity Test

Genomic DNA of previously identify CRE-48 and CRE-232, which harboured OXA-48 and OXA-232 genes, respectively, were extracted and quantified using a NanoPhotometer™ (Implen, Germany). The DNA was then 10-fold serially diluted and subjected to real-time PCR assay with HRM analysis.

Specificity test was performed on a panel of strains, which were comprised of 17 Enterobacteriaceae (*Serratia marcescens*, *Escherichia coli*, *Enterobacter aerogenes*, *Enterobacter cloacae*, *Klebsiella pneumoniae*, and *Citrobacter freundii*), 15 non-Enterobacteriaceae (Methicillin-resistant *Staphylococcus aureus* (MRSA), *Candida albicans*, and *Haemophilus influenzae*), and five *Acinetobacter baumannii* isolates with OXA-23 carbapenemase genes. The presence of OXA-48-like gene in the 17 *Enterobacteriaceae* was previously confirmed by PCR. Among the 17 *Enterobacteriaceae*, one *E. coli* isolate was confirmed to be OXA-232-producer based on sequencing data. All the isolates were revived and subjected to the developed assay.

### 4.5. Further Evaluation of the Assay Using the CRE Isolates Collected from January 2016 to December 2016

The developed assay was further evaluated using CRE isolates newly collected from the UMMC. In total, 45 isolates were collected within a duration of 12 months and the isolates were subjected to conventional PCR-sequencing and real-time PCR assay with HRM analysis for identification of the *bla*_OXA-48_ variants.

## 5. Conclusions

In conclusion, we have successfully developed a HRM analysis assay that is able to detect and distinguish OXA-48-like carbapenemase gene variants in a rapid and effective way. This assay requires little interpretation, and we believe that it will be important in OXA-48 carbapenemase gene dissemination control and epidemiology investigations.

## Figures and Tables

**Table 1:** Evaluation of high-resolution melting (HRM) analysis using carbapenem-resistant *Enterobacteriaceae* (CRE) isolates collected in 2016.

Organisms	Strains ID	Conventional PCR-Sequencing	HRM Analysis
*Klebsiella pneumoniae*	CRKP 30	OXA-232	OXA-232/OXA-181
*Klebsiella pneumoniae*	CRKP 37	OXA-232	OXA-232/OXA-181
*Klebsiella pneumoniae*	CRKP 38	OXA-181	OXA-232/OXA-181
